# Sexual and reproductive health services during outbreaks, epidemics, and pandemics in sub-Saharan Africa: a literature scoping review

**DOI:** 10.1186/s13643-022-02035-x

**Published:** 2022-08-09

**Authors:** Mwila Ng’andu, Aldina Mesic, Jake Pry, Chanda Mwamba, Florence Roff, Jenala Chipungu, Yael Azgad, Anjali Sharma

**Affiliations:** 1 Centre for Infectious Disease, Lusaka, Zambia; 2 University of Washington, Seattle, Washington, USA; 3 Avert, Brighton, UK

**Keywords:** Access, Utilization, Epidemics, Ebola, COVID-19, Adolescents, Young people, Family planning, Maternal health

## Abstract

**Background:**

The COVID-19 pandemic could worsen adolescent sexual and reproductive health (ASRH). We sought evidence on the indirect impacts of previous infectious disease epidemics and the current COVID-19 pandemic on the uptake of ASRH in sub-Saharan Africa (SSA) to design relevant digital solutions.

**Methods:**

We undertook a literature scoping review to synthesize evidence on the indirect impacts of COVID-19 on ASRH in SSA per the Arksey and O’Malley framework and PRISMA reporting guidelines. We conducted the search on PubMed, Embase, Google Scholar, and ResearchGate in June and November 2020. We included all peer-reviewed, English-language primary studies on the indirect impacts of infectious disease epidemics on the uptake of sexual and reproductive health (SRH) in SSA.

**Results:**

We included 21 of 42 identified studies. Sixteen studies (76.2%) quantitatively assessed utilization and access to SRH during epidemics. Five studies (2 [9.6%] qualitative and 3 [14.3%] mixed methods) explored factors affecting SRH services. All studies focused on adult populations, most often on labor and delivery (*n* = 13 [61.9%]) and family planning (*n* = 8 [38.1%]) outcomes. Although we sought out to assess all outbreaks, epidemics, and pandemics, the only relevant studies took place during the West African Ebola pandemic (*n* = 17 [80.9%]) and COVID-19 pandemic (*n* = 4 [19.0%]). One study (4.8%) highlighted adolescent-specific outcomes and condom use. Most studies found declined access to and utilization of facility delivery, antenatal care, family planning, and HIV care. One study noted an increase in adolescent pregnancies. However, other studies noted similar, or even increasing trends in access to and utilization of other SRH services (family planning visits; HIV diagnosis; ART initiation) during epidemics. Barriers to SRH uptake included factors such as a reduced ability to pay for care due to lost income, travel restrictions, and fear of infection. Supply-side barriers included lack of open facilities, workers, commodities, and services. Community-based peer delivery systems, telemedicine, and transport services improved SRH uptake.

**Conclusion:**

Access to SRH services during epidemics among adolescents and young people in SSA is understudied. We found that no studies focused on SRH outcomes of abortion, emergency contraception, sexually transmitted infections, or cervical cancer. To improve access to and utilization of SRH during pandemics, we recommend the following; in terms of research, key standardized SRH indicators should be included in routine data collection, routine data should be disaggregated by age, gender, and geography to understand gaps in ASRH service delivery, and additional rigorous epidemiological and social-behavioral studies should be conducted. On implementation, community-based peer delivery systems and telemedicine, internet-based, and other technological solutions may better reach adolescent and young people in SSA.

**Supplementary Information:**

The online version contains supplementary material available at 10.1186/s13643-022-02035-x.

## Plain English summary

Adolescents and young people face barriers to accessing sexual and reproductive health (SRH) services such as birth control, condoms, HIV/AIDS and sexually transmitted infection (STI) testing. Changes in health care, social policy, and household’s economic status due to infectious disease epidemics may further reduce access to SRH services by young people. We conducted a literature scoping review on the impacts of past epidemics on SRH to anticipate and mitigate the indirect impacts of COVID-19 on SRH among young people. We conducted a search of literature related to SRH services during infectious disease epidemics in sub-Saharan Africa (SSA) and found 21 studies. Included studies focused on adult populations with only one study specific to adolescents. Our review showed that utilization and access to labor, delivery, and antenatal services decreased dramatically during the Ebola outbreak with long-lasting detrimental effects. Barriers to care included increased costs of care, difficulty traveling distances due to lockdowns, fear of infection, and a lack of operating facilities, workers, supplies, and services. The evidence for adult populations suggests that adolescents and young people may face heightened challenges to accessing SRH services during epidemics which may lead to poor health outcomes. This review highlights key areas for future research programs and policies.

## Background

Globally, adolescents and young people (AYP) bear a disproportionate burden of adverse sexual and reproductive health (SRH) outcomes [1, 2]. Of the estimated 1.8 billion early adolescents and young people (aged 10-24) worldwide, 90% live in low- and middle-income countries (LMICs) [3]. In sub-Saharan Africa, early adolescents (aged 10–19 years) constitute a significant proportion (25%) of the total population [4]. AYP experience adverse SRH outcomes due to early sexual debut and marriage, risky sexual behavior including multiple sexual partnerships and insufficient condom/contraceptive use [5, 6]. Adolescent girls face additional vulnerabilities including violence by intimate partners and non-partners, early and unintended pregnancy, and sexually transmitted infections (STIs)/HIV [7, 8]. Restrictive policies, an absence of adolescent friendly SRH services, and other factors (cultural, societal, and religious) may inhibit utilization of SRH services by young people [9]. On an individual level, AYP may be unable to access care due to distance and a lack of income, or may be unwilling to due to stigma, shame, and misinformation [9, 10]. Although many national and international bodies have prioritized AYP health, improvements in SRH outcomes and access to related services have been limited [8].

The COVID-19 pandemic in Zambia and other LMICs, specifically prevention measures, are expected to exacerbate barriers to SRH services and contribute to poor health outcomes among AYP [11–13]. Prior studies have found that essential services decline during epidemics. The West Africa Ebola pandemic made a notable impact on services including a disruption of childhood immunizations, significant reductions in maternal health services, and declines in malaria care seeking, all of which may have collectively contributed to more deaths than the virus itself [14–17]. Public health measures in an epidemic such as quarantines, school closures, and reallocation of resources towards emergency services, compromise essential services, which are dependent on functional and accessible health facilities [14]. Fear of contracting the infection, restriction of movement, distrust, and violence/mistreatment may further prevent the availability and utilization of essential services [15–18]. Thus, public health crises such as the ongoing COVID-19 pandemic could exacerbate barriers to SRH services and worsen AYP health [11–13].

Prior observational and modeling studies suggest that essential health services may decline during the COVID-19 epidemic, resulting in larger negative impacts on morbidity and mortality [19–21]. We undertook a literature scoping review to identify and synthesize knowledge on the indirect impacts of epidemics on access to and utilization of SRH services by AYP in SSA to design appropriate digital solutions. This scoping review highlights knowledge gaps and evidence to inform research, programming, and policies in Zambia, and other LMICs, during the ongoing COVID-19 pandemic to improve SRH outcomes in this key population.

## Methods

We conducted a scoping review driven to systematically map the literature on SRH services for AYP during public health crises. We aimed to describe gaps in research to guide further research, program, and policy opportunities during the ongoing COVID-19 pandemic [22]. This review was conducted in accordance with the Preferred Reporting Items for Systematic Reviews and Meta-Analyses Extension for Scoping Reviews (PRISMA-ScR) checklist [12] and a widely used methodological framework for scoping studies: the Arksey and O’Malley Framework (2005). Further, we considered more recent specific recommendations for strengthening the framework in our review stages [23]. We applied Arksey and O’Malley’s five recommended stages of scoping reviews, as outlined below.

### Research study identification

We developed research questions to guide the scoping review. The objective of conducting this scoping review was to understand access and utilization of SRH services among AYP during health crises. However, due to lack of literature during initial searches, we expanded the study to include adult (≥ 25 years of age) populations. Our specific research questions were the following: (1) what is the landscape of access to, and utilization of, SRH services during COVID-19 and prior public health crises in SSA? (2) What factors have contributed to access to and utilization of SRH during COVID-19 and prior public health crises in SSA?

### Literature identification

We conducted a full systematic search of relevant indexed peer-reviewed publications from June 15 to 30, 2020. Given how quickly COVID-19 literature evolved, we conducted another search from November 24 to 30 in three academic databases: (1) PubMed/MEDLINE (National Library of Medicine); (2) EMBASE (Excerpta Medica dataBASE); (3) Google Scholar. In addition, we searched ResearchGate and reference lists of articles for additional relevant studies. Search terms included the following population: adolescents (10–19 years); young people (10–24); and the general population. SRH outcomes included sexual behavior; contraceptive use; pregnancy; labor and delivery; HIV/AIDS; STIs; and gender-based violence. In terms of context, the review included any studies that collected data during epidemics in SSA. The aim of this scoping review was to understand indirect impacts of public health crises, including outbreaks, epidemics, and pandemics. Direct relationships between public health crises and SRH were excluded from review. We have included the main search terms in Table 1.

### Study selection

Studies were included based on the inclusion and exclusion criteria highlighted in Table 2. Included studies were limited to English-language, peer reviewed publications that could be accessed via a library service with primary data (e.g., quantitative, qualitative). Commentary articles, grey literature, and any studies not reporting primary data (i.e., modeling studies, systematic reviews) were excluded. Two team members (AM and MN) independently and systematically searched for all articles in the three databases and in ResearchGate using the search terms and inclusion/exclusion criteria.

### Data presentation

All relevant search results were exported into an electronic spreadsheet to manage and ensure completeness. Two reviewers (AM and MN) screened titles and abstracts with literature identification rules to ensure articles met the inclusion criteria. After screening, a review of full-text articles was conducted independently by both members to ensure the article met pre-determined criteria for inclusion. Discordant determinations were resolved through discussion and did not need a third reviewer. Reasons for not including an article were documented.

**Fig. 1:**
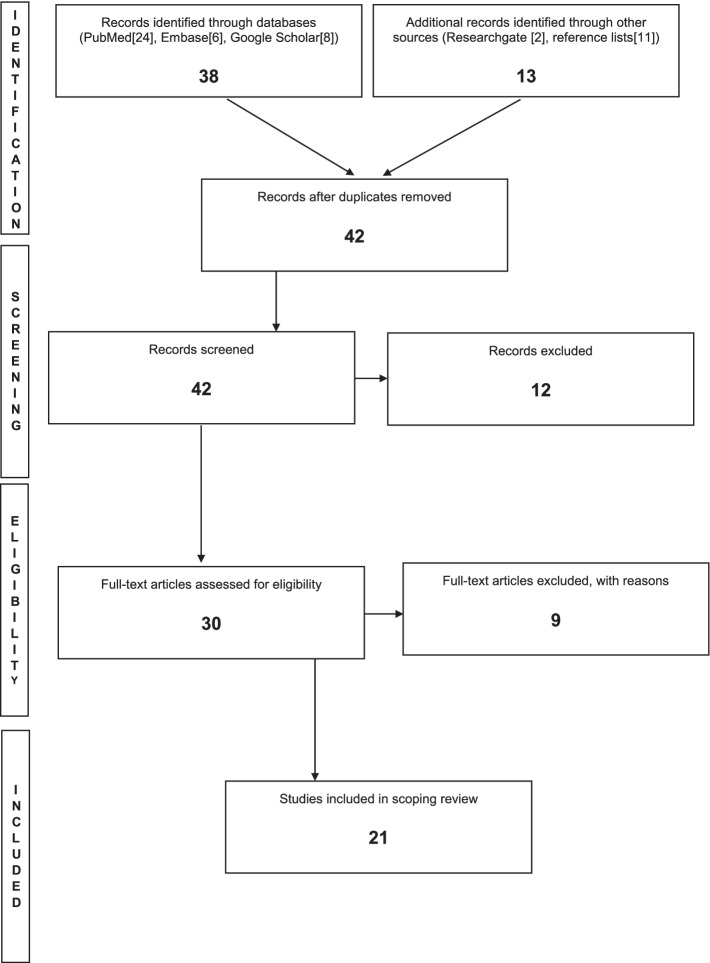
PRISMA study selection procedure flow chart

The results of the search are reported below and presented in a Preferred Reporting Items for Systematic Review and Meta-Analysis Protocols (PRISMA-P) flow diagram in Fig. 1*.*

After articles were selected, the reviewers independently conducted data extraction of the following variables: year; author; abstract; country/region; population/sample; type of study; type of data collected; key findings on utilization and access to SRH; factors (barriers and facilitators) related to SRH; and other interesting findings related to the research questions.

The team has summarized the findings below using the thematic areas that emerged through a priori themes informed by the research questions (i.e., deductive analysis) and those arising from the literature (i.e., inductive analysis).

## Results

### Scoping review results

We identified 51 relevant studies across PubMed, Embase, ResearchGate, and Google Scholar platforms, which met the search criteria for assessing SRH services during epidemics. After removing duplicates, 42 records remained, of which 12 were excluded due to inaccessibility or lack of relevance. Of the 30 full-text articles reviewed, 9 did not meet the inclusion/exclusion criteria described in Table 2. The final literature review included 21 peer-reviewed scientific papers across seven countries [24–44]. There were 17 articles on Ebola Virus Disease (EVD) covering three countries - Sierra Leone, Guinea, and Liberia [26–31, 33–43]. Four studies focused on COVID-19 and covered Ethiopia, Kenya, Nigeria, and South Africa [24, 25, 32, 44]. All studies investigated how epidemics impacted utilization and access to health services, including SRH services, with some studies also assessing general health outcomes (e.g., patient admissions and causes of death).

A summary of the included studies can be found in Table 3. All studies were published between 2015 to 2020 and used an observational design. Most studies focused on EVD (*n* = 17, 80.9%), and took place in West Africa (Guinea, Liberia, and Sierra Leone were the geographic focus in 5, 9, and 6 studies, respectively). The most studied outcomes in quantitative studies (*n* = 16) were labor and delivery (*n* = 12, 61.9%), family planning (*n* = 7, 33.3%), antenatal care (*n* = 6, 28.6%), and HIV (*n* = 6, 28.6%). Study details including authors, the epidemic, location, population, setting, data collection, and outcomes are included in Table 4.

### SRH access and utilization results

We have summarized the detailed results on access and utilization varied by SRH outcome in Supplementary Materials 1.

**Table 1:** Search terms^a^

Population	Adolescents, young people, adults, general population
Concept	Reproductive, sexual, contraception, family planning, contraceptive, HIV service, HIV testing, HIV program, HIV treatment, antiretroviral therapy, abortion, sexually transmitted infections, sexually transmitted diseases, morning after pill, emergency, cervical cancer screening
Context	Pandemic, epidemic, outbreak, COVID, COVID-19, coronavirus, Severe Acute Respiratory Syndrome Coronavirus-2 OR SARS-CoV-2.^b,c^

^a^The full search string included all variations of the search terms and associated acronyms

^b^The focus of this review was initially on adolescents and young people, but given very few relevant studies, the population was broadened

^c^We expected all relevant outbreaks (e.g., cholera, Ebola) but would be captured with terms such as “pandemic”, “epidemic” and “outbreak”

**Fig. 2:**
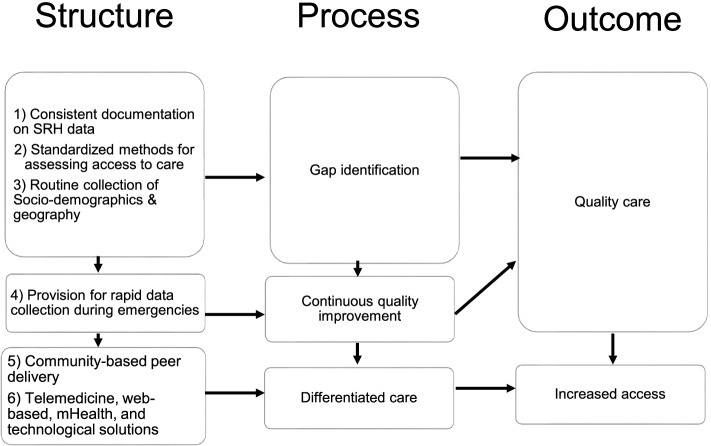
Recommendations for improving AYP health during pandemics within the Donabedian model

**Table 2:** Scoping inclusion and exclusion criteria

Criteria	Inclusion	Exclusion criteria
Publication type	Peer Reviewed; full text available through a library service	Not Peer Reviewed; full-text not accessible
Language	English	Non-English
Setting/place	Sub-Saharan Africa^a^	Not sub-Saharan Africa
Study design/type	Any studies with primary data (i.e., observational studies, randomized controlled trials, qualitative studies)	Commentaries; systematic reviews; meta-analyses; scoping reviews; modeling studies
Time limit	Any time	None

^a^According to the World Bank, sub-Saharan Africa includes the following countries: Angola, Benin, Botswana, Burkina Faso, Burundi, Cameroon, Central African Republic, Chad, Congo, Cote d'Ivoire, Eritrea, Ethiopia, Gabon, Gambia, Ghana, Guinea, Guinea-Bissau, Kenya, Lesotho, Liberia, Madagascar, Malawi, Mali, Mauritania, Mauritius, Mozambique, Namibia, Niger, Nigeria, Rwanda, Senegal, Sierra Leone, Somalia, South Africa, United Republic of Tanzania, Togo, Uganda, Zaire, Zambia, and Zimbabwe

**Table 3:** Description of studies included in the scoping review (*N* = 21)

Variable	Number of studies (%)
Sub-Saharan African countries	
Ethiopia	1 (4.8%)
Guinea	5 (23.8%)
Kenya	2 (9.5%)
Liberia	9 (42.9%)
Nigeria	1 (4.8%)
Sierra Leone	6 (25.6%)
South Africa	1 (4.8%)
Type of data collected	
Quantitative	16 (76.2%)
Qualitative	2 (9.6%)
Mixed methods/multi-methods	3 (14.3%)
Type of study	
Observational	21 (100%)
Year of publication	
2015	6 (28.6%)
2016	2 (9.5%)
2017	5 (23.8%)
2018	1 (4.8%)
2019	2 (9.5%)
2020	5 (23.8%)
Pandemic	
Ebola	18 (85.7%)
COVID-19	3 (14.3%)
Sexual reproductive health outcomes^a^	
Labor and delivery (L&D)	13 (61.9%)
Family planning (FP)	8 (38.1%)
Antenatal care (ANC)	7 (33.3%)
HIV	6 (28.6%)
Maternal mortality (MM)	4 (19%)
Condoms	1 (4.8%)
Adolescents and young people (AYP)	1 (4.8%)

^a^Only quantitative studies (*n* = 18) are included

**Table 4:** Studies on SRH care during pandemics included in the scoping review (*n* = 21)^a^

Study	Epidemic; location	Population/setting	Type of data collected; period of data collection	Sexual reproductive health outcomes
Abdela, 2020 [24]	COVID-19; Dessie town, South Wollo Zone, Ethiopia	Dessie referral hospital	Facility registers February 2–April 19, 2020	FP, ANC, L&D
Bietsch, 2020 [25]	Ebola; Liberia and Sierra Leone	Facility-level service statistics; DHS data	Pre-Ebola 2013 DHS data; service statistics quantitative electronic routine facility-level data; Survey data from Multiple Indicator Cluster Survey (MICS) 6 months before the first Ebola case and 37 months after last case of main outbreak in Liberia and Sierra Leone	FP, Condoms
Brolin Ribacke, 2016 [26]	Ebola; Sierra Leone	32 government, private, not- and for-profit healthcare facilities offering emergency obstetrics	Facility surveys and service-statistics captured using DHIS 2; Three periods between January 2014-May 2015: Pre-outbreak period (week 1–21, 2014), outbreak peak (week 22–52, 2014), and outbreak slow down (week 1–20 2015)	L&D
Camara, 2017 [27]	Ebola; Macenta District, Guinea	187,094 women of reproductive age (15-45 years)	Facility-level data Pre-Ebola (March 1, 2013- February 28th, 2014), intra-Ebola (March 1, 2014 to February 28th, 2015) and post-Ebola (March 1, 2016 to July 31, 2016)	L&D, ANC, MM
Delamou, 2017 [28]	Ebola; six districts in the Forest Region of Guinea	One regional hospital, five district referral hospitals, two community hospitals, 38 health centers, serving 1,747,4000 people	Facility-level data Pre-Ebola (January 2013- February 2014), during-Ebola (March 2014–February 2015) and post-Ebola (March 2015–February 2016).	L&D, ANC, AYP
Iyengar, 2015 [29]	Ebola; Margibi County and Bong County, Liberia	75 primary healthcare facilities in Margibi and Bond Counties	Service statistics from routine electronic facility-level DHIS-2 March–December 2014	L&D, FP
Jacobs, 2017 [30]	Ebola; Liberia	All individuals > 15 years included in the DHIS-2	Service statistics from routine electronic facility-level DHIS-2 Pre-Ebola (2013), during Ebola (2014), and post-Ebola (2015)	HIV
Jones, 2016 [31]	Ebola; Sierra Leone	13 comprehensive and 67 basic health care facilities across 13 districts	Data collected via facility surveys and facility registers	L&D, ANC, MM
Konwloh, 2017 [32]	Ebola; Liberia	All patients in Liberia with presumptive and active TB that were investigated, diagnosed, or treated between 2013 and 2015	Pre-Ebola (January 2013–March 2014), during Ebola (April 2014–June 2015) and post-Ebola (July–December 2015). Facility-level service statistics from DHIS2	HIV
Leuenberger, 2015 [33]	Ebola; Macenta District in the Forest Region of Guinea	Centre Medical, a specialized hospital, the only HIV care facility in the district	Routine and prospective facility-level data for hospital planning and reporting to health authorities; internal accountancy data, and data collected as part of the International epidemiological Databases to Evaluate AIDS (IeDEA) West Africa collaboration During Ebola (August–December 2014) and Pre-Ebola (August–December 2013) For retention in care, data was collected for a longer period (first semesters of 2013 and 2014 before Ebola)	HIV
Lori, 2015 [34]	Ebola; Bong County, Liberia	12 study sites from Bong County	Facility-level January 2012–October 2014	L&D
Loubet, 2015 [35]	Ebola; Liberia	5948 patients across two hospitals, John F. Kennedy and Redemption Hospital	Facility-level data Pre-Ebola (January 2012 to June 2014); point break to indicate during Ebola (June–November 2014)	HIV
Ly, 2016 [36]	Ebola; Rivercess County, Liberia	1,298 women from 941 households	Household survey Pre-Ebola (March 24, 2011–June 14, 2014), during Ebola (June 15, 2014–April 13, 2015)	L&D
McQuilkin, 2017 [37]	Ebola; 15 counties in Liberia	543 households were cluster sampled from catchment areas of 21 government hospitals	Household structured questionnaires March–May 2015	L&D, FP
Miller, 2018 ^a^ [38]	Ebola; Guinea (Dubréka, Forécariah, Macenta and Kérouané Districts), Liberia (Lofa, Montserrado, Margibi, and Bong), and Sierra Leone (Kenema, Kailahun, Bombali, and Tonkolili)	582 participants from the MoH UN agencies, iNGOs, NGOs, traditional healers, community leaders, caregivers of children under five, CHWs, TBAs, officers in charge of health facilities, MCH aides, members of CHCs and EVD survivors selected using purposive non-probability sampling	Routine program data from the MoH and NGO implementing partners January 2013 to December 2015 In-depth interviews and focus group discussions February–August 2016 (Liberia: February–March; Sierra Leone: May–June; Guinea; July–August)	
Quaglio, 2019 [39]	Ebola; Pujehun district, Sierra Leone	77 community health facilities and one hospital	Routine facility-level health services data Pre-Ebola (January 1, 2012–May 30, 2014), Ebola (June 1, 2014–February 28, 2015), Post-Ebola (March 1, 2015–December 31, 2017)	L&D, FP, ANC
Siedner, 2020 [40]	COVID-19; uMkhanyakude district, Kwa-Zulu Natal, South Africa	46,523 across 11 primary care clinics	Routine health facility data from HDSS and AHRI Pre-lockdown (January 27–March 27, 2020), level 5^c^ lockdown (March 28, 2020–April 30, 2020), level 4^c^ lockdown May 1-31, level 3^c^ lockdown until data abstraction date (June 1-30).	FP, ANC
Barden-O'Fallon, 2015 [41]	Ebola; All four geographic zones of Guinea (Upper, Lower, Middle, and Forest)	A convenience sample of 16 hospitals and 29 health centers that were categorized as “active”; “calm” and “not affected” in relation to Ebola cases; 62 health service directors; 117 RMNCH providers	Retrospective quantitative facility-level data collected from October 2013–December 2014 (categorized as Ebola Active, Changing Status, or Inactive) Brief structured qualitative interviews January-February 2015.^b^	L&D, FP, HIV, MM
Ahmed, 2020 [42]	COVID-19; Seven slums in Nigeria, Kenya, Pakistan, and Bangladesh;	*N*=860 purposively selected community leaders, residents, health workers, and local authority representatives	Qualitative data from individual discussions (20–50 min) and group discussions (1–3 h). Pre-COVID (March 2018–March 2020), as part of the *Improving Health in Slums Collaborative*, and during COVID (April-May 2020).^b^	L&D, HIV
Elston, 2016^a^ [43]	Ebola; Moyamba District in the Southern Region and Koinadugu District in the Northern Region, Sierra Leone	60 stakeholders including Ebola response teams, civil/transition authority, healthcare workers, members of NGOs, community members, a women’s group, mothers with children attending a child health clinic, social mobilizers and town council members 15 purposively selected health facilities in Moyamba	Interviews with 60 stakeholders Focus group discussions February and May 2015.^b^	
Gichuna, 2020 ^a^ [44]	COVID-19; Nairobi, Kenya	117 female sex workers 15 healthcare providers	Semi-structured interviews (15–20 min) over mobile phones April–May 2020.	

^a^Acronyms in Table 4: *AHRI* Africa Health Research Institute, *AYP* Adolescents and Young People, *CHC* Community Health Center, *CHW* Community Health Worker, *DHIS2* District Health Information Software, *DHS* Demographic Health Survey, *EVD* Ebola Virus disease, *FP* Family Planning, *HDSS* Health Demographic Surveillance System, *L&D* Labor and Delivery, *iNGO* international non-governmental agency, *IPTp* Intermittent Preventive Therapy for Malaria, *MM* Maternal Mortality, *MoH* Ministry of Health, *NGO* non-governmental agency, *PNC* Prenatal care, *ANC* antenatal care, *RMNCH* reproductive, maternal, newborn and child health, *SHRH* Sexual Health Reproductive Health, *TBA* Traditional Birth Attendant, *TT2* tetanus toxoid, UN: United Nations

^b^Findings not reported by outcome area for qualitative studies

^c^In South Africa, a level 5 order is considered a shelter in place order which includes closure of schools and non-essential businesses and restrictions on movement and public transportation. Residents were instructed to remain in their homes unless they were “performing an essential service, obtaining an essential good, or seeking emergency, lifesaving, or chronic care.” At the end of April, South Africa moved to level 4, then level 3 which lifted several restrictions. Level 4 allowed for some businesses and transportation to open. Level 3 included the opening of many establishments (e.g., cinemas, restaurants, gyms) and increased access to local and long-distance travel

**Table 5:** Summary of quantitative and qualitative barriers and facilitators affecting SRH utilization and access during pandemics (*n* = 7)

Barriers	Facilitators
Increased cost of medicines and supplies	Resources to alleviate travel difficulties
Difficulty traveling and long distance from facilities	Alternative modes of care delivery
Fear of infection from health facilities	
Lack trust in health system or quality of care provision	
Demographic factors such as not being educated	
Supply side issues including closure of health facilities, lack of workers, services, and supplies	
Stigma associated with infection	

#### Labor and delivery

Of the 18 studies (16 quantitative and 2 mixed methods), many showed a decline in facility deliveries in the Ebola period compared to the pre-Ebola period [28, 26, 30, 31, 33, 35, 38]. One study also showed a statistically significant increase in institutional deliveries in a rural district in Sierra Leone, potentially due to few Ebola cases, but a negative trend in the transition from Ebola to post-Ebola [43]. A study on COVID-19 showed that facility deliveries remained stable at the start of the COVID-19 epidemic in Ethiopia [24]. Complications such as gynecology emergency [24], pregnancy complications [26], major direct obstetric complications (MDOC) cases [43] and cesarean-sections [28, 31, 43] each decreased during Ebola compared to the post-Ebola period. Other studies showed that maternal admissions [43] as well as obstetric access [41] declined during outbreak periods.

#### Maternal mortality

Similarly, mixed findings on maternal mortality emerged with two studies showing an increase [26, 35] and one study showing a reduction in maternal deaths during Ebola with a significant increase after Ebola [43].

#### Antenatal care (ANC)

ANC services were dramatically reduced during the EVD epidemic compared to the pre-Ebola period [24, 31, 33]. This was consistent for ANC visit 1, 2, 3 or more visits [24, 26, 30]. The post-Ebola period saw a slight increase in ANC 1 and 3 visits compared to the intra-Ebola phase in Guinea [30].

#### Family Planning

Between pre- and post-Ebola periods, new and continuing family planning visitations increased in health centers but decreased in hospitals [26]. One study showed a decline in family planning consultations during the Ebola outbreak compared to pre-Ebola period in rural Sierra Leone [43]. During the COVID-19 outbreak, we found conflicting results across countries. While a study from rural South Africa reported an increase in daily clinic visitations for family planning [44], another reported that at a referral hospital in Dessie town, Ethiopia, family planning visits decreased by more than 95% after the implementation of COVID-19 precautions [24]. All the studies reported a decline in the utilization of all types of contraception during the EVD epidemic compared to the post-Ebola period. This included a stockout of modern contraceptives (i.e., injectables, pills, condoms) in most facilities [26] and a decrease in distribution of implants and contraception pills and the associated couple-years of protection (CYP) [27]. One study showed that the distribution of male condoms fell during the EVD epidemic to 22% compared to a pre-Ebola average of 51% [27].

#### HIV services

Two studies showed a decline in HIV-related facility visits in the Ebola period compared to the pre-Ebola period [37, 39]. However, Siedner (2020) reported an increase in HIV related visits with reduced COVID-19 restrictions [44]. HIV testing decreased during the Ebola outbreak compared to the pre-Ebola period across all the studies that reported on HIV testing [26, 34, 36, 37]. HIV diagnosis showed a significant decline in one study [36] while another showed similar trends in diagnosis between pre-Ebola and post-Ebola periods [34]. There was a significant drop in newly enrolled patients on ART in most of the studies [34, 37, 39]. While one study showed a decline in ART initiation among TB patients newly diagnosed with HIV in Liberia [36], analysis of Liberia’s DHIS data showed increased ART initiation among people presenting to healthcare facilities during and after the EVD outbreak [34].

#### Adolescents

As aforementioned, we identified a gap in the literature in regard to adolescent-specific studies. One mixed-methods study revealed significant increase in the mean teenage pregnancies per chiefdom in Moyamba district of Sierra Leone during the Ebola outbreak (173 pregnancies) compared to the pre-Ebola phase (137 pregnancies), *p* < 0.03 [31]. Respondents to qualitative interviews opined that since schools had closed, sexual activity particularly involving young girls and older men had increased. The authors cautioned that the apparent 25% increase in teenage pregnancy may be an underestimate given pregnancy requires clinical diagnosis (i.e., may be delayed if care-seeking is delayed) and because schools were subsequently closed again due to the outbreak.

### Results: barriers and facilitators related to access to and utilization of SRH during epidemics

Seven studies discussed barriers and facilitators affecting SRH utilization during epidemics. Table 5 summarizes barriers and facilities and Supplementary Materials 2 present detailed findings by study and factor.

#### Barriers

Across countries, the COVID-19 pandemic increased cost of medicines and supplies. A study noted that individuals working in the informal sector could not afford to buy medicine due to a lack of income after COVID-19 restrictions were imposed, while the health facility could not pay the higher costs of supplies [25]. Several studies noted an increased challenge in traveling to healthcare facilities, especially among those who lived more than 10 km away, or those affected by poor road conditions, limited transport, and movement restrictions [25, 32, 40, 42]. Fear of nosocomial infection prevented health service access and utilization across settings and populations for both COVID-19 and Ebola epidemics [25, 40, 41]. In a study of 15 counties in Liberia, nearly 60% of participants from rural areas and 24% from urban areas cited fear of Ebola infection as the major barrier to care seeking [41]. In other settings, many study participants did not trust the health system and believed circulating rumors that healthcare workers gave children the virus through immunizations [31]. Also, others stated that they did not believe that they would receive high quality care through the public health system [31, 42]. In addition to epidemic-specific barriers, socio-demographic factors such as low household wealth status and low maternal education were associated with decreased odds of facility delivery during Ebola [40].

Several supply-side issues affected healthcare access and utilization including healthcare facilities closing and/or reducing hours during the EVD and COVID-19 epidemics [25, 32, 41]. Other studies noted reductions in services including reproductive and maternal care, HIV testing, and delivery services [25, 26, 31]. Lastly, participants noted the reductions in contraceptive and pregnancy testing supply chains affected their ability to access them when needed during the COVID-19 pandemic [32].

#### Facilitators

Some health system responses demonstrated promising facilitators for increasing access to SRH services during epidemics. During the COVID-19 outbreak in Kenya, phone consultations and an emergency phone number to access free taxi transfers at night addressed transportation difficulties for pregnant women [25]. Similarly, the West African Ebola epidemic saw an increased use of traditional birth attendants, community health workers, and traditional healers for prenatal care, deliveries, and child services [41, 42]. However, while community health workers filled an important gap in SRH services, they did not receive the support they needed to ensure safe home deliveries or referrals to facility-based deliveries [41, 42].

## Discussion

Overall, this comprehensive scoping review revealed the scarcity of literature on SRH services during epidemics in SSA. The studies covered two pandemics (Ebola and COVID-19) though there have been several other disease outbreaks, such as influenza, bubonic plague, cholera, yellow fever, meningitis, measles, rift valley fever, and polio in SSA [45]. Similarly, the literature lacked variety in SRH outcomes. Nearly all studies assessed facility delivery, family planning, antenatal care, or HIV, with no studies evaluating sexually transmitted infections or cervical cancer screening, abortion care, or gender-based violence care. This paucity of information is particularly worrying given evidence from prior humanitarian crises that such care is essential to prevent unintended pregnancies, unsafe abortions, complications, intimate partner violence, and other adverse health outcomes [46–50]. Further, as noted, no studies focused exclusively on AYP’s access to and utilization of SRH, despite the alarms raised regarding heightened vulnerabilities of this population [1, 2].

Globally, governments are taking unprecedented measures to limit the spread of the COVID-19 virus, while health and social systems are struggling to cope with rising caseloads, supply chain bottlenecks, movement restrictions, and economic difficulties. In humanitarian/fragile settings and LMICs, where systems are already weak, the epidemic may cause more collateral and long-lasting damage without thoughtful and comprehensive SRH services. In a recent mathematical modeling study, Riley et al. (2020) estimated a 10% proportional decline in use of contraceptive methods in LMICs during the COVID-19 pandemic [19]. Across 132 LMICs, this reduced access would result in nearly 49 million women having an unmet need for modern contraceptives and 15 million women having unintended pregnancies over the course of a year during the COVID-19 pandemic (ibid). A 10% decline in service coverage would result in an estimated 1.7 million additional major obstetric complications and 28,000 maternal deaths [19]. Concerningly, these estimates do not take into account the increased risk of adverse health outcomes associated with adolescent pregnancies and births, which would likely mean even higher numbers and worse outcomes among adolescent girls. Further, other investigators have estimated that COVID-19 disruption could led to a 10% increase in HIV mortality, nearly 77,000 deaths in the next year [20, 21].

This review confirms that leaving SRH unaddressed amid a public health crisis impacts access and utilization during and after the epidemic. Many studies included in this scoping review showed that access to SRH services, notably facility delivery and antenatal care declined during the early and post Ebola outbreak phases in West Africa. However, some better-funded services such as HIV and family planning were more resilient. Methodological differences such as setting/sample (e.g., facility-based vs. national), analysis techniques (e.g., difference in difference, times series), and the number and types of SRH services create variability in observed magnitude and direction of impact. Health system context and temporality may also account for the observed differences. For instance, in Ethiopia, facility-level deliveries remained stable early in the epidemic, but gynecological emergency visits decreased; in Guinea health centers performing better than hospitals; and, in Sierra-Leone government facilities performed better than private, not-for-profit facilities during the peak, but worse during the slow-down of the Ebola epidemic [24, 26]. Furthermore, urbanicity could explain utilization, for instance, both fewer Ebola cases and higher SRH utilization was observed in rural Sierra Leone while an increased fear of nosocomial infections may have adversely affected SRH utilization in rural Liberia [41, 43]. Importantly, there are indications that fear of exposure and depleted resources (e.g., staff, supplies) limited the supply of services while the fear of nosocomial infection and loss of livelihood limited the demand for SRH services. However, under these circumstances, accessible, and trusted community healthcare workers met SRH needs, albeit with insufficient training and resources.

The findings from this scoping review led us to provide clear recommendations for SRH service delivery to AYP during pandemics as listed in Fig. 2 [51, 52]. Firstly, this review found that adolescent’s access to SRH services during epidemics have received little attention, as highlighted in the several knowledge gaps. This review highlights the need for studies to assess the unique needs, barriers, and facilitators which AYP may encounter during epidemics. Observational studies which can collect or leverage rapid data on utilization of SRH services for both AYP and the general population can inform localized responses. This data should be disaggregated by sex, age, and geography to further understand the heterogeneity in service delivery between sub-populations. This is particularly relevant for AYP, as numerous shortcomings in AYP health measurement have been identified. These include inconsistent indicators, poor harmonization with existing data, and data that is not well aligned to needs [53]. Relatedly, consistent documentation which enables real-time feedback and quality improvement can greatly improve access to and quality of services. Lastly, studies should aim to follow best practices in epidemiological reporting for observational studies (i.e., Strengthening the Reporting of Observational Studies in Epidemiology [STROBE]) [52]. We found that the reporting of methods and outcomes across studies was largely inadequate and varied greatly, making comparisons and generalizations challenging in this scoping review. These issues also limit the ability to conduct future, more rigorous reviews (i.e., systematic reviews, meta-analyses) which would have required an assessment of bias and commentary on the quality of the articles.

Beyond data and research recommendations, we have outlined two key areas which may improve AYP health during epidemics. Firstly, we recommend leveraging existing community-based peer delivery systems to increase access to prophylactics, contraceptives, and ART. Our scoping review found that formal healthcare utilization decreased across several outcome areas, while simultaneously home-based services (namely, deliveries) increased. This was likely due to both demand-side issues (i.e., fear of infection), and supply-side issues (i.e., closed facilities). This finding is well-aligned with other calls for increased demand-generation and community-based activities alongside existing facility-based offerings, to improve AYP SRH access [54]. Secondly, we recommend integrating telemedicine, internet-based, and other technological solutions to reach AYP. There is strong evidence to support the use of mobile Health (mHealth) programs targeting AYP SRH [55]. Prior mHealth interventions have aimed to increased knowledge sharing and behavior change and link AYP to essential SRH services. Given the widespread use of mHealth interventions in LMICs, there is an existing infrastructure which could potentially be used to build epidemic-specific mHealth interventions and reduce barriers to care for this key population, particularly during public health crises.

### Recommendations

1. Support consistent documentation and representation of key SRH data elements with real-time feedback to make quality improvements [51]
2. Reduce methodological heterogeneity in assessing access and utilization of SRH services during epidemics, using “Strengthening the Reporting of Observational Studies in Epidemiology” (STROBE) statement [52]
3. Disaggregate routinely collected SRH data by age, sex, and geography to understand gaps in service delivery to sub-populations during pandemics [51, 53]
4. Additional studies should be rapidly implemented to capture information on SRH services that are not routinely recorded
5. Leverage community-based peer delivery systems for prophylactics, contraceptives and ART/other chronic illnesses could increase access to essential services for AYP [54]
6. Telemedicine, internet-based and other technological solutions may be appropriate to reach AYP who may otherwise not have the means or the autonomy to access SRH services [55]

Figure 2 presents the Donabedian Structure-Process-Outcome (SPO) model [56], a conceptual framework to summarize and organize our recommendations to improve AYP health during pandemics. We postulate “structure” in terms of (1) consistent documentation and representation of key SRH data elements; (2) standardized methods to measure access and utilization of SRH services using the STROBE statement; (3) disaggregation of routinely collected SRH data by age, sex, and location; (4) provision for rapid data collection during emergencies (e.g., funding, scientific support, and swift ethical approval); (5) institution of community-based peer delivery systems for prophylactics contraceptives and ART/other chronic illnesses; and (6) development and implementation of telemedicine, mHealth, and other technological solutions for hard-to-reach populations that impact directly on “process”. The processes include continuous quality improvement (QI) based on real-time feedback, common understanding of unmet SRH needs by sub-populations (i.e., gap identification), and provision of services relevant to the unique needs of populations including adolescents (i.e., differentiated care). The structures and processes will contribute to increased access to and quality of SRH services.

This review had several limitations. We may have missed additional relevant studies through our inclusion of only peer-reviewed, English language, and full-text publications. For example, we did not have any studies related to the recent Ebola epidemic in DRC, which may have been a result of the peer-review inclusion criteria. Relatedly, though we searched for relevant articles several times throughout the study (in June and November 2020), some articles may not have been included in this analysis given how quickly and continuously the literature has evolved for COVID-19. Also, we did not assess the rigor or quality of these studies, indicating that this does not represent as rigorous of a process that would be expected for a systematic review. Most studies relied on routine facility-level data, which may have issues with data quality and completeness. Despite these limitations, our study highlights that SRH services will be disrupted and access to and utilization of services will decrease without deliberate efforts to address the needs of all seeking care, particularly AYP and adolescent females.

## Conclusion

Indirect effects of infectious disease public health crises can be long term. It is critical that support for access to and utilization of SRH services be maintained or, better still, improved during epidemics. Particularly, services which address the unique needs of AYP are markedly absent. Findings suggest that more data and research in SSA are needed to understand SRH access and utilization. Data should be disaggregated by age, sex, and urbanicity and account for methodological and cultural/contextual differences to quickly understand gaps and develop localized responses. Recommendations to improve AYP SRH access include leveraging existing community-based delivery systems and technological approaches to increase access, knowledge, and promote behavior change during epidemics. Without targeted efforts to improve access, adverse SRH outcomes will increase, reversing recent progress in SSA and LMICs.

## Supplementary Information

**Additional file 1: **Supplementary materials 1. Detailed quantitative Findings on Access and Utilization by SRH Outcome During Pandemics (*N*=18). Supplementary materials 2. Barriers and Facilitators Affecting Access and Utilization of SRH Services During Outbreaks, Epidemics and Pandemics

## Data Availability

Everything is included in the study tables and references.

## Abbreviations

ANC: Antenatal care
ART: Anti-retroviral treatment
ASRH: Adolescent sexual reproductive health
AYP: Adolescents and young people
CIDRZ: Centre for Infectious Disease Research in Zambia
COVID-19: Corona virus disease 2019
CYP: Couple years of protection
EMBASE: Excerpta Medica database
EVD: Ebola virus disease
FP: Family planning
LD: Labor and delivery
LMIC: Low-middle income countries
MDOC: Major direct obstetric complications
MeSH: Medical subject headings
MM: Maternal mortality
PRISMA-P: Preferred reporting items for systematic reviews and meta-analyses protocols
PRISMA-ScR: Preferred reporting items for systematic reviews and meta-analyses extension for scoping reviews
SARS-CoV-2: Severe acute respiratory syndrome coronavirus-2
SRH: Sexual reproductive health
SSA: Sub-Saharan Africa
STI: Sexually transmitted infections
STROBE: Strengthening the reporting of observational studies in epidemiology
