# Supplementary material for: Sexual and reproductive health services during outbreaks, epidemics, and pandemics in sub-Saharan Africa: a literature scoping review
Source: Syst Rev. 2022 Aug 9;11:161. doi: 10.1186/s13643-022-02035-x (PMC9361234; doi:10.1186/s13643-022-02035-x)
Supplement: Supplementary file 1 — Additional file 1: Supplementary materials 1. Detailed quantitative Findings on Access and Utilization by SRH Outcome During Pandemics (N=18). Supplementary materials 2. Barriers and Facilitators Affecting Access and Utilization of SRH Services During Outbreaks, Epidemics and Pandemics [file 13643_2022_2035_MOESM1_ESM.docx]

***Supplementary Materials 1: Detailed quantitative Findings on Access and Utilization by SRH Outcome During Pandemics (N=18)***

| **Labor and Delivery (n=13)** |
| --- |
| **Delivery**   - Facility delivery remained stable after COVID-19 precautions were implemented in Ethiopia (24). - Studies on the Ebola outbreak showed a decrease in institutional deliveries, with the exception of Quaglio, In Sierra Leone (2019) who show an increase (β 11; 95% CI 2-22) during this period (43). Deliveries decreased:   - In government facilities by 15% during outbreak peak and 36% during the outbreak slow down in Sierra Leone (28).   - In private, non-profit hospitals by 37% during the outbreak peak, and 5% during the outbreak slow down in Sierra Leone (28).   - Child births in health centres (HCs) and hospitals saw a 31% and 7% decline respectively in Guinea (26).   - There were 240 less women per month having institutional deliveries (95% CI −293 to −187) compared to pre-Ebola period in Guinea (30).   - In community HCs, the mean number of monthly deliveries reduced from 24.2 (95% CI: 19.2 –29.2, *p*<0.017) pre-Ebola to 13.8 (95%CI: 8.0 –19.6) during the outbreak in Sierra Leone (31).   - Facility delivery utilization in August 2014 was at 33% of the high in March 2014 in Liberia (33).   - In Sierra Leone, facility delivery significantly decreased to 11% (IRR 0.89, 95% CI 0.87 to 0.91) (35).   - Facility-based deliveries reduced to 113 in August and 160 in September from 587 in May in Liberia (38).   - 30% reduction in the odds of facility-based delivery (AOR=0.70, 95%CI 0.50–0.98, p = 0.037) (40).   - The mean number of institutional deliveries pre-Ebola (1203, SD=38, P<0.001) declined by 62% in the intra-Ebola period to 464 deliveries. Post-Ebola period saw an improved mean of 792 visits (SD=26) indicating a 34% significant drop in institutional deliveries compared to the pre-Ebola period (29). - With the exception of Quaglio, in Sierra Leone (43) who show a decrease (β -3; 95%CI -3 to -1), studies reported improved utilization during the post-Ebola period though not to pre-Ebola levels:   - Average of 149 women per month (95% CI 91–206) in Guinea (30).   - An average of 306 births in facilities compared to between 400-500 pre-Ebola in Liberia (38).   **Complications**   - Gynaecology emergency visits decreased in Ethiopia during COVID-19 (24). - In Guinea, complications in pregnant women declined by 20% between the 1st and 5th quarters (26). - In Sierra Leone, average MDOC increased post-Ebola (β 43, CI 31 to 54) compared to pre-Ebola but reduced in the transition from Ebola to post-Ebola (β −3, 95%CI −5 to −1, *p*=0.001) (43).   **C-sections**   - C-sections decreased by 5% in government and 50% in private, non-profit hospitals during the outbreak period in Sierra Leone (28, 2016). - Averages for C-sections increased post-Ebola from pre-Ebola in Sierra Leone (β 19, CI 13-25) (43). - After a declining trend during Ebola outbreak, there was a full recovery in C sections observed in the post-Ebola monthly mean of caesarean section (37, SD=8) as compared to the pre-Ebola level (38, SD=7, p=0.692) (29)   **Other**   - In Sierra Leone, mean monthly maternity admissions decreased from 46.4 (95% CI 38.3-54.5) to 25.8 (17.3-34.3), *p*<0.01 (31). - Among 358 women seeking obstetric care, 67% (238) accessed it; 77.7% (185) from urban areas, 22.3% (53) from rural areas (*p*< 0.001) (41). - Use of traditional birth attendants increased by 10% for Obstetrics and gynecological care during the epidemic (41). - In Sierra Leone, averages for maternal admissions increased post-Ebola (50, CI 37 to 64) compared to pre-Ebola but had a negative trend in the transition from Ebola to post-Ebola (−7, 95%CI −10 to −4) (43). - Decrease in maternity waiting homes (MWH) use from 84 in May to 27 in August 2014 in Liberia (38).  \| **Family Planning (n=8)** \| \| --- \| \| **New family planning visits**   - In Guinea, the percentage of family planning acceptors in Health Centers (HC) increased by 36% between the pre-Ebola compared to post-Ebola period while in hospitals, a 37% decline in family planning acceptors was experienced between the pre-Ebola compared to post-Ebola period (26).   **Any family planning visits**   - Family planning visits decreased by more than 95% due to COVID-19 precautions in Ethiopia (24). - From 531 mean visits (SD=35) in the pre-Ebola period, family planning visits saw an increase of 47% in the early months then later declined to 242 visits in the peak month of the outbreak, (a 51% drop from pre-Ebola levels). The post Ebola era saw 98% recovery in family planning visits with a mean of 503 visits (SD=66, p=0.573) with similar patterns observed across different family methods used, except for implant and intrauterine devices (IUD) (29)   **Continuing family planning visits**   - Health Centers experienced increased continuing family planning by 6% between the pre- and post-Ebola period in Guinea, whilst hospitals experienced a 17% decrease between the pre- and post-Ebola surveyed period (26). - Resilience in family planning visits observed in South Africa, increasing from 7.3 visits/clinic/day in the pre implementation (pre-lockdown) period to 7.8 visits/clinic/day after transition to level 5 (+0.5 visits/clinic/day, 95%CI−1.0 to 2.0) to 8.9 clinic visits/day after transition to level 4 (+1.1 visits/clinic/day, 95% CI −0.7 to 3.0) and 11.0 visits/clinic/day after transition to level 3 (+2.0 visits/clinic/day, 95% CI 0.3 to 3.7) for a 66% total increase from the pre lockdown period (44).   **Contraceptive**   - In Guinea 21% of the facilities reported a shortage of at least one type of modern contraception (injectables, oral pills or condoms) in the pre-Ebola compared to post-Ebola period although only 5% reported that this stockouts occurred during the Ebola outbreak from April 2014 to December 2014 (26) - In Liberia, distribution of implants during the Ebola crisis fell to 20% of pre-Ebola levels. On average, implant distribution was down 43% from pre-Ebola distribution during the outbreak. About 19 to 24 months post-Ebola saw an increase of 25% to 75% in implant distribution compared to the 6 months prior to the Ebola outbreak (27). - In Sierra Leone implants had the sharpest decline, falling to a minimum of 70% of their pre-Ebola levels. Post-Ebola saw an increase in implant insertions up to 70 percent at 19–24 months post-Ebola. Due to the increase in the number of implants towards the end of the crisis, the average number of implants inserted throughout the crisis was nearly the same as the average distribution in the 6 months before the epidemic (27). - In Liberia, injectables fell to half the pre-Ebola levels in their lowest month and averaged 83% of pre-Ebola levels over the course of the epidemic. The post-Ebola period saw substantial growth in injectables by 77% compared to pre-Ebola levels. In Sierra Leone, injectables declined to between 75–80 percent of their pre-epidemic levels, averaging 84–90 percent of pre-epidemic distribution over the course of the epidemic and was lower on average 19–24 months post-Ebola compared to the average over the 6 months pre-Ebola (27). - In Liberia, pill distribution declined to 43% of pre-Ebola levels in its lowest month, but over the period of the epidemic averaged 81% of pre-epidemic levels. Post Ebola period pill distribution increased by 16% compared to pre-Ebola levels. While in Sierra Leone, oral contraceptive pill distribution declined to between 75–80 percent of their pre-epidemic levels, averaging 84–90 percent of pre-epidemic distribution over the course of the epidemic was lower on average 19–24 months post-Ebola compared to the average over the 6 months pre-Ebola (27).   **Other**   - During the Ebola crisis, couple-years of protection (CYPs) fell to an average of 7,999 per month, growing to from 11,794 in the first 6 months to 16,012 during months 19–24 post-crisis, 39% above pre crisis levels. The average monthly distribution of contraception was 10,424CYP in Liberia, increasing by an average of 275 CYPs per month; and 15,794 in Sierra Leone (27). \| \| **Antenatal Care (n=7)** \| \| **Antenatal Care Visits**   - The number of pregnant women coming for antenatal care decreased by more than 50% in Ethiopia (24). - In Sierra Leone, in November 2013, 324 women =>15 years old attended the community health centre compared to 1 person in November 2014 (31). - In Bong County and Margibi county in Liberia, antenatal care visits were 14% and 9% of peak utilization during the lowest months of utilization during the Ebola epidemic (33). - An 18% reduction in ANC visits was observed after the onset of the Ebola outbreak in Sierra Leone (IRR 0.82, 95% CI 0.79 to 0.84) (35) - During the Ebola outbreak, 418 less women per month were going for at least one ANC visit (-418, 95% CI −535 to −300) compared to pre-Ebola period, increasing to an average of 173 more women per month post-Ebola (95% CI 51–294; *p*=0·0074) in Guinea (30). - Pregnant women seen for 1st ANC visit in surveyed health centres declined by 13% between the pre-Ebola and post-Ebola period in Guinea (26). - In Sierra Leone, negative trends pre-Ebola versus post-Ebola were observed for ANC 1 (−6, 95%CI −10 to −3) (43). - Third trimester visits declined by 16% between the 1st and 5th quarters in Guinea (26). - During the Ebola outbreak, 363 less women per month went at least three ANC visits (-363, 95% CI −485 to −242) than pre-Ebola, increasing to an average of 257 more women per month post-Ebola (95% CI 51–294) in Guinea (30). - ANC4 utilization appeared to have declined post-Ebola compared to pre-Ebola period (−8, 95%CI −11 to −5) (43). - In Sierra Leone, negative trend in differences for ANC 4 observed pre-Ebola versus post-Ebola (−85, 95%CI −119 to −51) (43). - ANC visits all showed a similar trend as they declined by 59% from a monthly mean of 2053 visits (pre-Ebola) to 842 visits (intra-Ebola). Post-Ebola era experienced an increase of 1260 mean visits (SD=21) compared to intra-Ebola but showed a 37% significant decline compared to pre-Ebola (1987, SD=34, p<0.001) (29)   **Prenatal Care Visits**   - Reduction in the number of PNC visits by 22% (IRR 0.78, 95% CI 0.75 to 0.80; *p*<0.001) in Sierra Leone (35). - Of 361 women seeking prenatal care, 66% (236) accessed it; 77.1% (182) from urban areas AND 22.9% (54) from rural areas (*p*<0.001) (41).   **Other**   - There was an increased use of traditional birth attendants (10%) for Obstetrics and gynaecological care during the epidemic, and more prenatal patients seeking care from traditional healers (41). \| \| **HIV Care (n=6)** \| \| **HIV Care Visits**   - In Guinea, there was a 11% drop in the number of follow-up visits at the HIV clinic but with no clear month-by-month pattern from August to December 2014 compared to the same period of 2013 (37). - The proportion of HIV follow up visits per week slightly increased between January 2012 and June 2014 pre-Ebola period (*p*=<0.001) which decreased by 50-59% during the Ebola crisis (*p*<0.05) in Liberia (39). - Adult HIV-related clinical visits/clinic/day increased between the pre lockdown and level 5 lockdown period in South Africa (increase of 7.9, 95%CI 2.1 to 13.8), between level 5 and level 4 lockdown (increase of 11.1, 95%CI 4.1 to 18.0) or in transition from level 4 to level 3 (increase of 4.0, 95%CI −2.5 to 10.5) (44).   **HIV Testing**   - HIV testing among pregnant women in surveyed hospitals showed a 51% decline between the surveyed quarters (*p*<0.05) in Guinea (26). - In Liberia, the median individuals per county tested annually for HIV declined by 35% (2444/6930) during the Ebola outbreak. Number of people testing for HIV dropped by 41% from 207,314 before Ebola to 121,952 during the outbreak. Post-Ebola 130,881 people had HIV tests, a 36.8% decline from pre-Ebola levels (34). - Proportion of TB patients tested for HIV declined from 72% pre-Ebola to 69% intra-Ebola and 68% post-Ebola *p*=<0.05 in Liberia (36). - HIV tests done and positive HIV test results dropped by 46% and 53%respectively between August and December 2014 (intra-Ebola) compared to the same period of 2013 (pre-Ebola) in Guinea (37).   **HIV Diagnoses**   - In Liberia, the proportion of HIV-positive remained similar in pre and intra Ebola periods (3.4% vs 3.5%) (34). - The proportion of TB patients tested for HIV showed a significant decline of 15% pre-Ebola, 14% intra-Ebola and 12% post-Ebola period (*p*=<0.05) in Liberia (36).   **Newly Enrollment in HIV Care/ART**   - While 61% (4311/7020) of newly diagnosed HIV patients were enrolled in care before the Ebola outbreak, the proportions during and after the outbreak were respectively 63% (2617/4143) and 76% (3501/4588, *p*<0.001) in Liberia (34). - About 33% (2,350/7,020) of newly diagnosed HIV patients were started on ART before the Ebola outbreak, this proportion increased to 43% (1787/4143) during and 53% (2,434/4,588, *p*<0.001) after the outbreak in Liberia (34). - The proportion of TB patients tested for HIV and on ART showed a significant decline of 34% pre-Ebola, 30% intra-Ebola and 26% post-Ebola period (*p*<0.05) in Liberia (36). - Mean number of visits per week reduced from 203 and 207 to 140 and 85 in two clinics during the Liberia Ebola outbreak. New patients per week significantly decreased in both clinics by 57% and 4.6% per week respectively (*p*< 0.001) (39). - There was a 47% drop in new enrolments of HIV-infected patients into chronic care at the Centre Médical clinic, Guinea, from August to December in 2014 compared to 2013 (37). \| \| **Maternal Mortality (n=4)** \| \| - Deaths among pregnant women sharply increased by 100% in hospitals between the surveyed periods in Guinea (26). - In Sierra Leone, the facility-based maternal mortality ratio increased by 34% after onset of the Ebola epidemic (1RR 1.34, 95% CI 1.07-1.69) (35). - In Sierra Leone, average maternal deaths reduced during Ebola compared to pre-Ebola times (−1, 95%CI -2 to 0), with a slight significant increase from Ebola to post Ebola period (2, CI 1 to 3) (43). - Maternal deaths were low and remained similar across the three periods (0.1–0.2%, p>0.05). Adverse newborn outcomes (deaths and stillbirths) were also low across the periods (range 1.1–1.7%) but higher in the post-Ebola period as compared to the pre-Ebola period (p<0.01) (29). \| \| **Condoms (n=1)** \| \| - In Liberia, post Ebola, male condom distribution fell to 83% of pre-Ebola outbreak levels. Throughout the epidemic, minimum and average male condom distribution fell to 22% and 51% of pre-Ebola levels, respectively. In Sierra Leone, male condoms distribution declined to between 75–80 percent of their pre-epidemic levels, averaging 84–90 percent of pre-epidemic distribution over the course of the epidemic and was lower on average 19–24 months post-Ebola compared to the average over the 6 months pre-Ebola (27). \| \| **Adolescents (n=1)** \| \| - Mean teenage pregnancy increased in Sierra Leone, from 137.6 (95% CI 96.4 –178.9) per Chiefdom in 2013 to 173.1 (117.1 –229.2) in 2014, *p*<0.03 (31). \|  1. Acronyms in *Supplementary Materials 1:* CYP: Couple-years of protection, HC: health center, MDOC: Major direct obstetric complications   ***Supplementary Materials 2: Barriers and Facilitators Affecting Access and Utilization of SRH Services During Outbreaks, Epidemics and Pandemics***   \| **Barrier** \| \| \| --- \| --- \| \| Increased cost of medicines and supplies \| - Before the COVID-19 pandemic, the cost of buying medicine was a problem for many residents in Nigeria and Kenya as they depended on informal sector employment, and therefore, paying for medicine became more challenging during the pandemic when informal sector employment was restricted/reduced (25). - Cost of many health-related items, including facemasks, hand sanitizers, disinfectants, gloves and drugs, especially those bought from private providers initially increased in Kenya (25). \| \| Difficulty traveling and distance from facilities \| - In Guinea, Liberia, and Sierra Leone, distance from health facility increased challenges posed by poor road conditions, limited transport, and the cost of transport (42). - Due to increased challenges in reaching health care facilities for pregnancy-related emergencies, a slum resident in Kenya and Nigeria might first call a traditional birth attendant who sometimes, if necessary, would accompany patients to hospital (25). - In Liberia, the adjusted odds of facility-based delivery were 15% lower during Ebola if a woman lived less than 10km away from the facility (AOR 0.85, CI 0.78–0.92, *p*<0.001). Distance from the facility in kilometres did not change meaningfully across its different levels (<10km, 10 to 21km, >21km) (40). - In Kenya, sex workers described being stuck in areas that did not have their required services, such as antiretrovirals (32). \| \| Fear of infection from health facilities \| - In Liberia, 56.0% (95%, CI 51.5–60.4) reported the belief that health facilities posed a risk of EVD transmission. Those who believed health facilities to be an Ebola transmission source had fewer facility-based deliveries (AOR = 0.60, 95% CI 0.36–0.99) than those who did not report this belief (OR = 1.03, 95% CI 0.63–1.70) (40). - Nearly 60% of respondents in rural areas and 24% in urban areas cited fear of contracting EVD in health-care facilities as the major barrier to accessing care (41). - Fear of contracting Ebola from health facilities was evident as Ebola was frequently associated with health facilities in Sierra Leone (31). \| \| Lack trust in health system or quality of care \| - In Sierra Leone, community members felt let down by the health system and believed that it could not address the needs and problems of the people and saw no point in going to health facilities (31). - In Sierra Leone, many people blamed the health system for the outbreak and suggested that it was responsible for the outbreak and was giving children the virus through immunisations. Such rumors in their communities had created a societal pressure to refrain from bringing children to the clinics (31). - In Guinea, Liberia, and Sierra Leone, a perception of poor-quality care provided at the health facility also caused community members to be reluctant to seek care outside the community (42). \| \| Demographic factors \| - The odds of facility-based delivery were 30% lower during the Ebola period when adjusted for maternal education (AOR 0.70, CI 0.50–0.98, *p*=0.037). In unadjusted analyses, secondary or higher education compared to no education increased the odds of a facility-based delivery (OR 2.40, CI 1.37–4.20, *p*=0.003) in Liberia (40). \| \| Supply side issues including closure of health facilities, lack of workers, services, and supplies \| - In Liberia, about 35% of respondents in urban areas cited closure of health-care facilities as the largest barrier to accessing care (41). - Reduced hours meant that adolescents and sex workers did not have adequate time to interact during the Safe Space or get peer support in Kenya (32). - In Nigeria and Kenya, there were reduced opening hours of health facilities, stock of medicine/supplies, and staff members due to lockdowns during the COVID-19 pandemic (25). - In Nigeria and Kenya, there was reduced availability of preventative services at health facilities, including reproductive and maternal health care. Further, services delivered by community health workers were stopped due to COVID-19 lockdown in Kenya (25). - In Liberia, nearly 32% of respondents claimed that HCWs refused to give care (41). - In Guinea, 6% of 62 directors said HIV tests and delivery services were suspended due to Ebola; 28% of 117 RMNCH service providers reported increases in complications due to delays in seeking delivery and maternal care (26). - There were reported stock outs of HIV drugs and disengagement of HIV patients from services during Ebola in Sierra Leone (31). - In Kenya, the production of contraceptives has been disrupted during COVID-19, and multiple participants indicated that they were not able to get pregnancy test kits or family planning (32). \| \| Stigma associated with infection \| - In Nigeria and Kenya, slum residents were reluctant to go to a hospital outpatient clinic for symptoms such as cough and fever for fear of being suspected of having COVID-19 (25). \| \| Facilitators \| \| \| Resources to alleviate travel difficulties \| - In Kenya, an emergency phone number that would provide free taxi transfer to health facilities at night was set up during the COVID-19 outbreak (25). \| \| Alternative modes of care delivery \| - In Nigeria and Kenya, health workers across sites reported using phones to consult with other colleagues to provide advice to pregnant women from a distance (25). - In Liberia, the use of traditional birth attendants increased (10%) for obstetrics and gynaecological care during the EVD epidemic, and more prenatal patients sought care from traditional healers. However, this did lead to a drop in facility-based care (41). - In Guinea, Liberia, and Sierra Leone, caregivers appeared well informed about CHW services and reported that they would first seek care from a CHW if their child was ill (42). - In Guinea, Liberia, and Sierra Leone, most stakeholders engaged in this study stated CHWs were willing to continue their regular activities during Ebola, motivated by a desire to contribute to “a cause larger than themselves” (42). - In Guinea, Liberia, and Sierra Leone, TBAs reported an increase in the number of home deliveries. However, these often took place without adequate materials or training (42). \|  1. Acronyms in *Supplementary Materials 2:* EVD: Ebola Virus Disease, AOR: Adjusted Odds Ratio |
